# Rheumatic Musculoskeletal Diseases and COVID-19 A Review of the First 6 Months of the Pandemic

**DOI:** 10.3389/fmed.2020.562142

**Published:** 2020-10-09

**Authors:** Martin H. Stradner, Christian Dejaco, Jochen Zwerina, Ruth D. Fritsch-Stork

**Affiliations:** ^1^Department of Rheumatology and Immunology, Medical University of Graz, Graz, Austria; ^2^Department of Rheumatology, Hospital of Brunico (SABES-ASDAA), Brunico, Italy; ^3^Trauma Centre Meidling, Ludwig Boltzmann Institute of Osteology at Hanusch Hospital of Oesterreichische Gesundheitskassa and Allgemeine Unfallversicherungsanstalt, First Medical Department Hanusch Hospital, Vienna, Austria; ^4^Medical Faculty, Sigmund Freud Private University, Vienna, Austria

**Keywords:** COVID-19, SARS-CoV-2, rheumatic and musculoskeletal disease, rheumatoid arthritis, systemic lupus erythematosus, Kawasaki disease, hydroxychloroquine, IL-6

## Abstract

In December 2019, a cluster of severe pneumonia was observed in China, with the subsequent discovery of a new beta-coronavirus (SARS-CoV-2) as the causative agent. The elicited disease COVID-19 is characterized by fever, dry cough, myalgia, or fatigue and has a favorable outcome in the majority of cases. However, in some patients COVID-19 leads to severe pneumonia and sepsis with subsequent respiratory failure and gastrointestinal, hematological, neurological, and cardiovascular complications. A higher risk of infection is intrinsic to active rheumatic and musculoskeletal diseases (RMD) and the use of biological disease modifying anti-rheumatic drugs (DMARDs). With an increasing number of reports on COVID-19 in RMD patients, we are beginning to appraise their risks. In this review, we summarize the published cases of COVID-19 infections in RMD patients, including patients with inflammatory arthritis and connective tissue diseases as well as anti-phospholipid syndrome and Kawasaki syndrome. Overall, patients with inflammatory arthritis do not seem to be at a higher risk for infection or a severe course of COVID-19. Risk for critical COVID-19 in patients with systemic inflammatory diseases such as SLE or vasculitis might be increased, but this needs further confirmation. Furthermore, we summarize the data on DMARDs used to fight SARS-CoV-2 infection and hyperinflammation.

## Introduction

Since the discovery of the novel coronavirus SARS-CoV-2 causing COVID-19 in December 2019 until June 2020, more than 30,000 reports on the disease or the virus itself have been published. Infections of patients with underlying rheumatic and musculoskeletal diseases (RMD) experiencing a complicated course of COVID-19 have been reported. However, most of these reports are limited by the small numbers of patients. Besides, several studies are still at a pre-publication stage and may therefore not yet meet the rigorous standards of scientific journals. This article summarizes current knowledge on COVID-19 and RMD. It also reviews the general risk of viral infections in patients with RMD, the impact of disease modifying anti-rheumatic drugs (DMARDs) on the outcome of infections, and gives a comparison between present and previous coronavirus pandemics.

## SARS-CoV-2 and COVID-19

The first cases of this novel pneumonia were reported in early December 2019 in Wuhan, the capital city of Hubei province, China (1). The genomic characterization of the pathogen causing this infection identified a novel enveloped positive single stranded RNA beta-coronavirus, which has ultimately been named severe acute respiratory syndrome coronavirus 2 (SARS-CoV-2) (2).

Coronaviruses are RNA-viruses, shielded by an envelope containing membranous proteins such as spike proteins. These spike proteins give the virus a crown like appearance under an electron microscope, coining the name “coronavirus.” The primary function of these proteins is host receptor binding, determining host tropism and transmission capacity (3).

Coronaviruses can cause a broad range of mostly mild infections in humans, mammals, and birds especially in the respiratory and gastrointestinal tracts, and to a lesser extent also in the nervous system. Until the end of 2019, six coronavirus strains (belonging to the alpha or beta-coronavirus genus) causing human disease had been identified (4). Four of these viruses cause common cold symptoms, whereas the two remaining strains elicit severe illnesses, namely the severe acute respiratory syndrome coronavirus (SARS-CoV) and the Middle East respiratory syndrome coronavirus (MERS-CoV) (4). Whereas, SARS-CoV was the pathogen leading to the severe acute respiratory syndrome observed in 2002/2003 in Guangdong Province, China (5), MERS-CoV was found to underly the severe respiratory syndrome seen since 2012 in the Middle East (6). Phylogenetically, the present SARS-CoV-2 displays an 88% homogeneity to two bat-derived severe acute respiratory syndrome (SARS)-like coronaviruses, a 79% identity with SARS-CoV and an approximate 50% identity with MERS-CoV (7). An important similarity between SARS-CoV and SARS-CoV-2 is the mechanism of entry into cells of the lower respiratory tract, mediated by the spike protein binding to the ACE2 protein (8).

The novel disease, termed COVID-19, is marked by symptoms reminiscent of other respiratory infections: fever, dry cough, myalgia, or fatigue are commonly observed, whereas sputum production, headache, hemoptysis, and diarrhea are less prevalent (1, 9). While the majority of patients with COVID-19 have a favorable outcome (10–13), some develop severe pneumonia eventually leading to acute respiratory distress syndrome (ARDS), respiratory failure, along with other organ manifestations and sepsis (14). COVID-19 appears to have at least two distinct disease phases: a phase characterized by the immune response against the virus with the aim of eliminating the pathogen. Some patients, even though they may have had only mild initial symptoms, subsequently develop a phase of severe “cytokine storm” (cytokine release syndrome, CRS) instead of the expected phase of re-convalescence leading to fatal autoinflammation of the lung and other organs (15). As the disease spreads throughout the world causing the present pandemic crisis, additional symptoms have been observed, e.g., neurological changes such as anosmia or ageusia or thrombotic complications (16, 17). The development of profound thrombocytopenia (36.2%) and elevated D-dimers (46.4%), which are even higher in patients with severe COVID-19 disease (57.7 and 59.6%, respectively) (9) have become a hallmark of the disease, and suggest a triggered disseminated intravascular coagulation (DIC), reviewed in (18). There are distinctive characteristics of this new disease: it affects more men than women, causes severe disease especially in elderly people. Certain comorbidities including hypertension, diabetes, prior respiratory, and cardiovascular disease as well as obesity have been associated with a worse outcome (9, 19). Based on a developing cohort of 1,590 patients and a validation cohort of 710 patients, a risk score for the development of a critical illness (defined as composite measure of admission to the intensive care unit, invasive ventilation, or death) 10 items emerged as independent predictive factors (12). Three of those were patient characteristics: older age, number of comorbidities, and a history of cancer. However, although immunodeficiency was one of the 72 potential predictors in the beginning, the presence of rheumatic diseases or immunosuppressive medication was not included in the calculation, making the score less applicable from a rheumatological perspective.

According to the first report of the Chinese Center for Disease Control only ~5% of patients out of 72,314 Chinese patients were critically ill, 14% of them suffered from a severe course, while most patients (81%) showed no or mild pneumonic symptoms (10). The overall case-fatality rate (CFR) was 2.3%, which is lower than that during the earlier coronavirus outbreaks with SARS-CoV (9%) and MERS-CoV (36%) (4). Whereas, the comorbidities and risk factors for worse outcomes of COVID-19 are similar throughout international reports, mortality rates diverge largely from 0.06% in Singapore up to 18.3% in France (13). The causes for this disparity are myriad and may include disease factors and genetic susceptibility of the populations, but also differences in health care organization (e.g., number of tests conducted, criteria for testing) and outcome reporting (particularly who is counted as deceased due to/with COVID-19).

Immunological changes generally observed in patients with COVID-19 include lymphocytopenia that pertains to CD4+, CD8+, and Treg cells and increased levels of proinflammatory cytokines [e.g., interleukin (IL)1-β, IL-1RA, IL-7, IL-8, IL-9, IL-10, interferon (IFN)γ, interferon-gamma induced protein (IP)10, monocyte chemoattractant protein (MCP)1, macrophage inflammatory protein (MIP)1α, MIP1β, tumor necrosis factor (TNF) α, granulocyte colony stimulation factor (GCSF), platelet derived growth factor (PDGF) B, and vascular endothelial growth factor (VEGF) A]. A pronounced decrease of CD4+ T cells and higher levels of IL-6, IL-8, and TNFα occur during the phase of hyperinflammation (CRS) (1, 20). Hyperinflammation is considered to initially involve activation of macrophages stimulated by damage-associated molecular patterns (DAMPs) released from dying cells and pathogen-associated molecular patterns (PAMPs) such as viral RNA (21). This leads to massive IL-1 and IL-6 secretion promoting the recruitment of neutrophils and cytotoxic T cells, which in turn induce pneumocyte and endothelial injury, ultimately leading to acute lung injury. Pre-publication reports describe a specific monocyte population in COVID-19 patients, not seen in healthy donors or other viral infections. These monocytes are larger, atypically shaped, vacuolated secreting high levels of both IL-6 and IL-1β and expressing ACE2 (22, 23). Interestingly, interferon type I, a crucial player in the combat of viral infections, seems to be muted for unknown reasons in COVID-19 patients (24), which was especially seen in critically ill patients (25). Furthermore, alterations in the lymphocyte department, including upregulation of markers of T cell exhaustion in both lung-resident and circulating T-lymphocytes, including PD-1 and Tim-3 have been described (26). Furthermore, CD4+ as well as CD8+ T cells of COVID-19 patients display an activated phenotype. In analogy to patients infected with SARS-CoV or MERS-CoV, CD4 Th1-cells expressing GM-CSF, and IL-6 were isolated from patients with COVID-19 CRS (27). In contrast, in patients in the recovery phase an effective adaptive immune response characterized by T cell clonal expansion, activation, and memory formation has also been reported in COVID-19 (23). The higher occurrence of the above described Th1 cells and the COVID-19-typical, highly inflammatory monocyte subset together with the inadequate IFN I answer may be determining factors leading to a worse disease course.

## The Infection Risk of Patients With Rheumatic Diseases

Patients with RMD are generally considered to be more prone to bacterial and certain viral infections such as the herpes zoster virus. The susceptibility to infections is multifactorial and is related to disease aspects such as disease activity, disease damage, comorbidities, as well as to treatment. In rheumatoid arthritis (RA), each 0.6 point increment of the disease activity score (DAS) (28), led to a 25% increase in hospital admissions and 4% increase of outpatient infections in a US registry of >16,000 patients (28).

A 6-fold increased susceptibility to infections was seen in systemic lupus erythematosus (SLE) in an insurance database (29) and an incidence rate of 29.2 severe infections/1,000 patients per year in the Spanish SLE-registry (30). Current and past use of HCQ seemed to have a protective effect (RR 0.49, *p* = 0.0000), whereas glucocorticoids (≥10 mg/d), rituximab, mycophenolate mofetil/mycophenolic acid, and lupus activity/severity were linked to a higher risk of severe infection.

## Susceptibility to Infections Through Immunosuppression

According to a Cochrane review, the risk of infection in patients with inflammatory arthritis [IA; including RA, psoriatic arthritis (PsoA) and spondylarthritis (SpA)] is higher under therapy with TNF-blockers than with conventional disease modifying anti-rheumatic drugs (DMARDs) only (31). A meta-analysis of other biological DMARDs (bDMARDs) used in RA indicated a 1.3-fold increased risk of serious infections in standard doses, and an almost 2-fold increased risk at higher doses (any dose higher than the one approved by the regularities) compared to conventional DMARDs (32). Whereas, the risk of serious infections under anti-IL-17 therapy is comparable to that of TNF-blockers; anti-IL-12/23 therapy seems to carry a lower risk (HR = 0.59, 95% CI 0.39–0.90) (33).

Due to their inhibition of interferon-alpha, Januskinase inhibitors (JAKi) increase the risk of viral infections (34), especially of herpes zoster. In contrast, the overall risk for severe infections of RA patients with JAKi is comparable to that of healthy controls according to a recent meta-analysis (35). Whether there is an increased risk of coronavirus infection (or influenza) elicited by JAKi used in rheumatology is currently unknown.

The use of glucocorticoids (GC) in both IA (36) and connective tissue diseases (CTD) leads (29) to an increased rate of infections overall and viral infections in particular, especially for herpes zoster correlating with the actual GC dose, treatment duration, and the cumulative GC dose; however even doses considered relatively safe such as 5 mg prednisone equivalent have been associated with an increased infections risk. Hydroxychloroquine seems to have a protective effect for those who take GCs: in patients with GC monotherapy a HR 3.9 for severe infections was reduced to 0 for those who took GC + hydroxychloroquine.

Among conventional synthetic DMARDs (csDMARDs), methotrexate has not been associated with an increased infection rate according to a study with over 27,000 RA patients (37). In contrast, mycophenolate mofetil, azathioprine, and cyclophosphamide were linked to a higher risk of infections particularly in patients with connective tissue diseases (38).

## Rheumatic Diseases and COVID-19

We know little of RMD patients infected in the past outbreaks of coronaviruses like SARS and MERS (39, 40). Also, given the restricted geographical spread and the low number of patients affected, no special containment measures were released affecting diagnosis and the management of patients with RMD, nor were there any difficulties with drug supply for DMARDs. There has been a weak association of coronavirus outbreaks in the past with higher incidence of Kawasaki syndrome, which is often virally triggered. Kawasaki syndrome occurred in <5% of patients with a coronavirus infection in some reports, however, no reports are available for SARS and MERS (41, 42). Single cases of SARS and thrombotic complications have been reported. These complications were considered to be related to the presence of anti-phospholipid antibodies (43).

### Occurrence of Rheumatic Diseases in Patients of Large Cohorts of COVID-Patients

In the largest early report on patients with COVID-19 from China there are no data on patients with RMD or any other immunological disorder (10, 44). Guan et al. mention two patients with immunodeficiency without a severe course of COVID-19 infection out of a cohort of 1,099 COVID-19 patients from China (9). Another publication from Wuhan included one patient with pre-existing connective tissue disease, who died (45). The cohort of 5,700 COVID-19 patients from New York City did not list any rheumatologic disease as a comorbidity, although at least CTD should have been captured, given that the Charlson comorbidity index was used in this study (19). Likewise, no RMD was named under the comorbidities in a prospective cohort of 1,150 critically ill patients in New York City or a cohort of 2,070 COVID-19 cases in Brazil (46, 47). In a paper from Lombardy, the Italian region with the highest number of COVID-19 cases (48), there were no patients with immunosuppressive therapy with JAKi or bDMARDs among 700 COVID-19 patients with a severe course of disease.

However, data from the health analytics platform OpenSAFELY, which covers 40% of all patients in England and holds primary care records of 17,278,392 adults pseudonymously linked to 10,926 COVID-19-related deaths found a hazard ratio of 1.19 (1.11–1.27) for the combined autoimmune diseases rheumatoid arthritis, lupus, and psoriasis (49).

Additionally, Arentz et al. reported 21 patients in intensive care units in Washington State. Of these, one person had a “rheumatic disease” as a pre-existing condition and three had immunosuppression due to “rheumatoid disease” or transplantation. However, there is no detailed information about the precise diagnosis, medication, comorbidities, or course/outcome of these patients (50). In that specific cohort, it seems that there was a high rate of co-morbidities (85.7%) and mortality (67%) with only a few discharges (9.5%).

Currently available data do not imply a strikingly increased risk of infection for RMD with or without DMARD therapy in general (51, 52), although differences in the individual diseases have been reported (53). Pablos et al. investigated the prevalence of COVID-19 in seven Spanish hospitals providing medical care for a population of 2.9 million patients, and found a comparable prevalence of the infection in RA and PsoA with the general population (0.58%); in contrast, patients with spondylarthritis (SpA) had a higher prevalence (53). As this was also the case for patients on bDMARDs or JAKi, one might speculate that the higher infection rate of SpA patients was due to a higher percentage of these drugs in SpA patients compared to the other groups. This aspect, however, was not analyzed in the cited paper. Interestingly, patients with Sjögren's syndrome or systemic sclerosis showed a higher prevalence of SARS-CoV-2 infection in comparison with the general population; in contrast, prevalence in SLE patients was similar to that of the reference population. This unexpected discrepancy among CTD patients might be explained at least partly by the younger age and the higher proportion of females among SLE patients in this cohort.

In a report from Lombardy, 1,525 patients with RMD were evaluated by a survey and 8% were found to have either confirmed or suspected COVID-19, with most of them suffering from a form of inflammatory arthritis (54). In addition, the authors used a case-control design to investigate differences between the 117 COVID-19 cases with RMD and control COVID-19 patients without RMD. No differences pertaining to comorbidities or disease course were detected.

Similar findings were described in a case control study from Massachusetts investigating 52 RMD patients, of which 50% had an IA and the remaining patients suffered from different forms of systemic autoimmune diseases. The disease course of COVID-19 of these cases was compared with that in 104 sex- and age-matched controls without underlying RMD (55). The majority of the RMD patients with COVID-19 had active disease (62%) and a higher percentage of coronary and interstitial lung disease. Whereas, the clinical presentation and laboratory values were similar in the two patient groups, RMD patients were at a higher risk for intensive care/mechanical ventilation [adjusted OR for mechanical ventilation 3.11 (95% CI 1.07–9.05), *p* = 0.04]. However, this did not imply a statistically significant higher mortality (6%) or overall hospital admission rate; the higher rate of ventilation reported in RMD patients is unsettling, considering that the OR was adjusted for the higher cardiorespiratory comorbidities, suggesting an intrinsic effect of the underlying autoimmune disease. Nevertheless, other comorbidities, disease activity or use of immunosuppressive drugs may have contributed to the higher risk of ventilation and need to be addressed in future studies. Similarly, a report from Wuhan found an increased rate of respiratory failure (38%; mortality: 9.52%) in 21 patients with RMD with a similar distribution between IA and CTD (56). In comparison with this Chinese study, a different cohort of 29 RMD patients from Wuhan found a lower need for ventilation (6.9%) and a 3.4% mortality rate (57). Whereas, the disparity of the first Chinese study with the cohort from the US may be attributable to a different disease population, different medication regimes, and also a different genetic background, the reason for the intra-regional difference between the two reports from Wuhan remains elusive. Altogether, there is a dire need for reports with larger cohorts.

To address this need, the Global Rheumatology Alliance has established a registry of RMD patients with COVID-19 infection (https://rheum-covid.org/). This is an international initiative, supported by ACR and the EULAR as well as by several national societies, with the possibility to include RMD patients affected by COVID-19 from all over the world.

The website is updated regularly (summarized in Box 1, accessed on July 19th 2020) and a report of the first 600 patients was recently published (58). The majority (93%) of patients were from North America or Europe, female, and between 50 and 65 years of age. The distribution of IA vs. CTD was approximately 2:1 and 20% displayed moderate/high disease activity. The report described interesting findings:

1. there was a high rate of hospitalization (46%) and mortality (9%) altogether, with SLE and vasculitis patients showing a higher propensity to be hospitalized than other patients.
2. the main factor associated with hospitalization was a prednisone dose ≥10 mg/day (OR 2.05, 95% CI 1.06–3.96), whereas DMARD therapy (csDMARD monotherapy or combined with bDMARDS/JAKi) was not associated with hospitalization.
3. therapy with TNF-blockers reduced the risk of hospitalization (OR 0.40, 95% CI 0.19–0.81) in contrast to therapy with antimalarials, which did not (OR 0.94, 95% CI 0.57–1.57).
4. established general adverse characteristics/comorbidities as age >65 years, hypertension/cardiovascular disease, lung disease, diabetes as well as chronic renal insufficiency/end-stage renal disease carried a higher risk of hospitalization also in RMD patients.
5. treatment with bDMARD/JAKi monotherapy just prior to COVID-19 diagnosis reduced the risk of hospitalization compared with no DMARD therapy (OR = 0.46, 95% CI 0.22–0.93; *p* = 0.03).

Box 1 **Data of patients from the rheum-covid registry, assessed on July 19 2020.**

- Patients in registry: 3,814
- Patient-characteristics on 1,440: 74.4% female, 76.2% <65 years, 39.5% Caucasian
- Outcome: death 8.06%, hospitalized 41.4%

RMD are distributed as follows:

- Rheumatoid arthritis (RA): 38.5%
- Systemic lupus erythematosus (SLE): 17.2%
- Psoriatic arthritis (PsA): 9.7%
- Vasculitis: 6.7%
- Spondylarthritis (SpA): 6.0%
- Sjögren's syndrome: 4.5%
- Systemic sclerosis: 3.1%

Top comorbidities:

- Arterial hypertension
- Lung diseases
- Diabetes
- Cardiovascular diseases
- Renal disease
- Morbid obesity

Medication prior to COVID-19:

- csDMARDs 62.8%
- bDMARDs 31.1%
- JAKi 4.9%
- CQ/HCQ 26.7%
- GC 33%

csDMARDs, conventional synthetic disease modifying antirheumatic drugs; bDMARDs, biological disease modifying antirheumatic drugs; JAKi, Januskinase inhibitors; CQ/HCQ, Chloroquine/Hydroxychloroquine; GC, glucocorticoids.

Some results, like the role and category of comorbidities, are in line with the general population experiencing COVID-19, others, like the protective effect of TNF-blockers were unexpected. The possible benefit of TNF-blockers was also observed in a cohort from the US (59) and underscores the role of the overabundance of TNF in critically ill COVID-19 patients. Altogether, this report supports the recommendations issued by national and international societies not to withhold DMARD treatment in RMD patients prophylactically in this pandemic. However, important questions as, e.g., a possible difference in disease course in the diverse RMDs have not been answered and await clarification.

The registry has also provided preliminary data on 80 SLE-patients with COVID-19 and the use of HCQ. Sixty-four percent of the SLE-patients were taking antimalarials prior to the infection with SARS-CoV-2 and admission frequency to the hospital did not differ between HCQ users and non-users [55% (16/29) vs. 57% (29/51), *p* = ns; χ^2^-test] (60). This argues against a protective role of HCQ (in the usually administered dose for RMD patients) in SARS-CoV-2 infection, which is also supported by pharmacological *in vitro* data describing a much higher level needed for effective viral inhibition (61). The ineffectiveness of HCQ was also indicated in several RMD and SLE cohorts, in which the prevalence of (confirmed or suspected) COVID-19 was similar in patients with or without HCQ treatment (62–68). In contrast, a large study using databases of general medication use and SARS-CoV-2 infection in Portugal found a protective effect of chronic treatment with HCQ [OR 0.51 (0.37–0.70)] for SARS-CoV-2 infection, even after adjustment for demographic characteristics and immunosuppressive treatment (69). Due to the study design, no data on underlying diseases (including RMD) or other patient data apart from those on medication use and COVID-19 status were retrievable precluding a definite judgement on the efficacy of HCQ for preventing COVID-19. In particular, a selection bias with prescription of HCQ preferably to patients without comorbidities could have influenced the results.

Other descriptions of RMD patients with SARS-CoV-2 infections are often based on case reports or case series (see Tables 1, 2 for summary) with different RMD and are discussed below.

**Table 1:** COVID-19 in patients with rheumatological diseases: inflammatory arthritis (IA).

**References**	**Disease (manifestation)**	**Patient number**	**DMARD**	**State of rheumatic disease**	**Treatment**	**Disease course**	**Comment (comorbidities)**
Song et al. (70)	RA	1	LEF, HCQ GC (2 mg)	Remission	Antiviral: L-R	Hospitalization	LEF and GC were paused
Duret et al. (71)	SpA	1	aTNF, 20 mg MTX	ns	PCM iv	Hospitalization	ns
Baba et al. (72)	RA	1	Iguratimod	ns	Inhalation with GC	Hospitalization	Prolonged presence of viral RNA
Cai et al. (73)	RA	1	LEF, HCQ	ns	Oseltamivir phosphate, L-R, MP (40 mg), 2x TCZ	ICU-admission resolved	Recurrent relapses of COVID-19 upon tapering of GC
Lee and Lee (74)	AS	1	aTNF, MTX	Remission	ns	Mild disease, not hospitalized	
Valenti et al. (75)	PsA	1	aTNF	ns	Antibiotic treatment (*n =* 1)	Hospitalized	Hypertension (*n =* 1)
Schulze-Koops et al. (76)	RA	2	RTX (*n =* 2) MTX (*n =* 2)	Remission (*n =* 1), ns (*n =* 1)	Antibiotic treatment (*n =* 1)	Both patients died	[COPD (*n =* 1), hypertension (*n =* 1)]
Brito et al. (77)	Behcets' disease RA SpA	1 1 1	aTNF aTNF aTNF	Remission Remission Remission	PCM PCM Azi	Mild symptoms, uncomplicated course, no hospitalization	(None)
Quartuccio et al. (78)	SpA (*n =* 3), RA (*n =* 1)	4	aTNF (*n =* 3), JAKi (*n =* 1), MTX *n =* 2)	Ns	Lopinavir/ritonavir (*n =* 3), ribavirin (*n =* 1), HCQ (*n =* 1)	Hospitalization: 75% Full recovery: 100% None ICU 2 mild disease, 1 moderate, 1 severe	[Latent Tbc (*n =* 2), hypertension (*n =* 1), chronic heart failure and atrial fibrillation (*n =* 1)]
Queiro Silva et al. (79)	PsA (*n =* 3) peripheral SpA (*n =* 1)	4	APR (*n =* 2), aTNF (*n =* 2)	Ns	ns	Hospitalized: 50% ICU: 50%,1 no symptoms, 1 moderate, 2 severe	(1 patients on ICU: hairy cell leukemia)
Cheng et al. (80)	RA (*n =* 4) SSc (*n =* 1)	5	RA: aTNF + MTX (*n =* 1), no DMARD (*n =* 2), LEF (*n =* 1) SSc: GC (8 mg/day for 1 year)	Ns	Arbidol (*n =* 5), ribavirin (*n =* 5), oseltamivir (*n =* 2) IFN-r (*n =* 3), antibiotics (*n =* 4), GC (*n =* 2), IvIG (*n =* 1)	Hospitalization: 100% ICU: 20% 2 with mild, 2 with severe course Mild and severe not further specified	[Hypertension (*n =* 3), previous Tbc + bronchiectasia (*n =* 1) COPD (*n =* 1)]
Favalli et al. (81)	RA (*n =* 3) SpA (*n =* 2) Sarcoidosis (*n =* 1)	6	aTNF (*n =* 5), ABA (*n =* 1), MTX (*n =* 2), LEF (*n =* 1), SSZ (*n =* 1), HCQ (*n =* 2)	Ns	ns	Hospitalization: 75% All resolved	Incidence: 0.62% in RMD comparable with general population [hypertension (*n =* 3)]
Monti et al. (48)	RA, SpA	4 SARS-CoV-2 positive (+ 4 suspected)	aTNF, TCZ, ABA, JAKi	Remission	Antibiotics in SARS-CoV-2 pos. patients Antiviral in hospitalized patient	Hospitalization: 12.5%	(Hypertension)
Sanchez-Piedra et al. (82)	RA (*n =* 21) SpA (*n =* 12) other RMD (*n =* 8)	41 (31 with positive PCR)	aTNF (*n =* 18), JAKi (*n =* 7), anti-IL-6 (*n =* 5), anti-IL-17 (*n =* 5), RTX (*n =* 3), Anakinra (*n =* 1), ABA (*n =* 2) MTX (*n =* 17), HCQ (=4)	Last DAS-28 mean 3.6 (SD 1.4)	ns	Hospitalization: 68% ICU: 14.6% Mortality: 7% Fully recovered at time of analysis: 85%	Charlson index mean 2.6 (SD 2.0), Hypertension (36.6%)
Haberman et al. (59)	RA, PsoA, SpA among non-rheumatological diseases	Total 86 patients, of which 54 with positive PCR 37 PCR pos RMD patients	71% of total patients bDMARD or JAKi	ns	Diverse, in hospitalized patients: HCQ + Azi: 10 HCQ/Azi/L-R: 3 HCQ/Azi/L-R/TCZ: 1	Hospitalization: 16% (of total patients) 1 patient died (on arrival to Hospital) 1 patient on ICU with ARDS	aTNF and anti-IL12/23 more often used in ambulatory patients
Fredi et al. (54)	RA (31%) PsoA/SpA (17%) SLE (10%) SSc (7%) Vasculitis (13%)	117 (confirmed and suspected)	GC: 59% HCQ: 19% csDMARD: 58% bDMARDs: 44%	ns	GC, TCZ in 23% Antivirals: 73% HCQ: 92%	Hospitalization: 40% (mostly RA) Thrombotic events: 15% Ventilation: 62% Mortality: 10% (75% of deceased had IA)	No difference in comorbidities, mortality and clinical/laboratoryparameter between COVID cases with or without RMD

*ABA, Abatacept; APR, Apresmilast; aTNF, anti-TNF agents; CAD, coronary artery disease; CKD, chronic kidney disease; GC, Glucocorticoids; HCQ, Hydroxychloroquine; ICU, intensive care unit; JAKi, Janus Kinase inhibitors; LEF, Leflunomide; MP, Methylprednisolone; L-R, Lopinavir/Ritonavir; TCZ, Tocilizumab; MTX, Methotrexate; PCM, Paracetamol; PIP/TAZ, Piperacilline and Tazobactam; PsoA, Psoriatic arthritis; RA, Rheumatoid arthritis; RTX, Rituximab; SSc, systemic sclerosis; SZP, Sulfasalazine; SpA, Spondylarthritis*.

*ns, not specified*.

**Table 2:** COVID-19 in patients with rheumatological diseases: connective tissue diseases (CTD).

**References**	**Disease (manifestation)**	**Patient number**	**DMARD**	**State of rheumatic disease**	**Treatment**	**Disease course**	**Comment (comorbidities)**
Mihai et al. (83)	Systemic sclerosis (ILD and arthritis)	1	TCZ	Quiescent	ns	Telephone monitoring at home	TCZ paused (IDDM, Obesity)
Avouac et al. (84)	Systemic sclerosis (skin, ulcera, arthritis)	3	RTX MTX: 66%	Normal Ig, but complete B cell depletion NO lung disease or PAH	1 patient: Anakinra, TCZ, GC All patients: antibiotics	Hospitalization: 100% Non-invasive ventilation: 100% Mortality: 0%	Late clinical worsening possibly by RTX
Guilpain et al. (85)	Granulomatosis with polyangiitis (ENT, orbital, lung, joint, and skin)	1	RTX, 15 mg GC (Previous: 41 g Cy), aTNF, MMF, MTX, LEF, RTX, GC	Relapse 4 months before: RTX induction Day before COVID-19: 500 mg RTX maintenance	Antivirals and HCQ	ARDS Mechanical ventilation Recovered	(Hypertension; BMI: 27)
Tomelleri et al. (86)	Large vessel vasculitis	4 (2 with TA, 2 with GCA)	Low dose GC	Remission	TA: no therapy GCA: HCQ	Complete recovery: 100% hospitalization: 50% mortality: 0%	(2x hypertension; 1x CAD and CKD)
Han et al. (87)	SLE	1	7.5 mg GC	ns	Antiviral Interferon nebulization PIP/TAZ	Admission to hospital	Diagnosis 7 weeks after presumed infection
Guven et al. (88)	SLE	1	ns	ns	ns	cMR: leptomenigeal micronodular lesions	No neurological symptoms reported
Romão et al. (89)	SLE	2	HCQ: 100% MTX: 50%	Remission	None	Complete recovery: 100% Hospitalization: 0% mortality: 0%	
Wallace et al. (67)	SLE/other RMD	5/31	HCQ: 80% CG: 80%	4/5 in remission	ns	Hospitalization: 80% Invasive ventilation: 60% Mortality: 20%	80% African American, 80% obese
Bozzalla Cassione et al. (63)	SLE	12	GC: 33% HCQ: 83% IS: 67%	ns	ns 1 patient on ICU: ventilation and MP	Hospitalization: ns Mortality: 0%	Prevalence COVID: confirmed: 2.5% suspected: 5% (Higher than general population)
Mathian et al. (62)	SLE (53% nephritis, serositis, 82% arthritis, 59% skin)	17	GC: 71% HCQ: 100% IS: 41%	16/17: quiescent	53%: antibiotics No antivirals 1 Patient TCZ	Respiratory failure: 65% ARDS: 29% Mortality: 14%	IS paused
Gartshteyn et al. (90)	SLE (61% nephritis, 5.5% hemodialysis, 11% kidney transplants)	18 (confirmed or high suspicion)	GC: 39% HCQ: 72%	Hospitalized patients: SLEDAI 8 Others: SLEDAI <4	HCQ (when not already taken) Antibiotics: 59% 43% of hospitalized patients: high dose MP and TCZ	Hospitalization: 39% Non-invasive ventilation: 11% Invasive ventilation: 5.5% Mortality: 0%	Overrepresentation of Hispanics (59%) and African Americans (39%) Possible protective effect of HCQ (43% of hospitalized vs. 91% of ambulatory patients on HCQ)
Zen et al. (91)	AAV, IIM, RA, SLE, SScl,	AAV: 182 IIM: 50 RA: 111 SScl: 176 SLE: 397	Ns	ns	2 hospitalized: 1 patient no treatment 1 patient (on MMF): Azi /HCQ	1 COVID-associated symptoms: 16% Suspicion of COVID: 2.5% Mortality: 0%	Infection rate comparable to general population
Favalli et al. (64)	SLE, SScl, SS, UCTD	SLE: 61 SScl: 43 SS: 10 UCTD: 9	BEL/RTX/TCZ: 20% IS: 60% GC: 64% HCQ: 25%	ns	ns	1 patient (SSCl with HCQ/RTX): fatal pneumonia 11%: respiratory symptoms (w/o SARS-CoV-2 test) Rapid resolution	Incidence of COVID-19 in RMD (0.81%) comparable to general population; no benefit of HCQ
Favalli et al. (92)	SLE	62	BEL: 42% HCQ: 48% GC: 74% RTX: 10%	ns	ns	13%: respiratory symptoms (w/o SARS-CoV-2 test) Rapid resolution	
Gendebien et al. (65)	SLE	225 confirmed or suspected COVID-19: 8% pos. PCR: 2%	BEL: 2% IS: 31% GC: 25% HCQ: 68% RTX: 1%	ns	ns	Hospitalization: 0.9%	No benefit of HCQ, no effect of IS on hospitalization, GC dose associated with pos. PCR, hospitalization, anosmia/ageusia, and diarrhea
Holubar et al. (93)	SLE	120/113 in survey high suspicion of infection: 7%	GC: 42% HCQ: 70% IS: 52.5%	ns	ns	Hospitalization: 0.9% Mortality: 0.9% (NOT SARS-CoV-2 associated)	No benefit of HCQ

*AAV, Anca associated Vasculitis; aTNF, TNF-blockers; Aza, Azathioprine; Azi, azithromycin; BEL, belimumab; CAD, coronary artery disease; CKD, chronic kidney disease; cMR, cerebral magnetic resonance; Cy, Cyclophosphamide; DVT, deep vein thrombosis; ELT, eltrombopag; ENT, Ear, nose, throat; ETA, Etanercept; GC, Glucocorticoids; GCA, Giant cell arteritis; HCQ, Hydroxychloroquine; IDDM, insulin dependent diabetes; Ig, Immunoglobulins; IIM, idiopathic inflammatory myositis; ILD, interstitial lung disease; IS, Immunosuppressant; IVIG, intravenous immunoglobulins; LEF, Leflunomide; MTX, Methotrexate; MMF, Mycophenolate Mofetil; PAH, pulmonary hypertension; PIP/TAZ, Piperacilline and Tazobactam; RA, Rheumatoid Arthritis; RTX, Rituximab; SAPS, secondary antiphospholipid syndrome; SS, Sjögren's syndrome; SScl, Sysetemic Sclerosis; SLE, Systemic lupus erythematosus; SLEDAI, systemic lupus erythematosus disease activity; TA, Takayasu arteritis; TCZ, Tocilizumab; UCTD, undifferentiated connective tissue disease; w/o, without*.

*ns, not specified*.

### Patients With Inflammatory Arthritis (IA) and COVID-19

The disease course in a series of 86 patients with immune-mediated inflammatory diseases including 50 cases of IA (rheumatoid arthritis, psoriatic arthritis, ankylosing spondylitis) with confirmed or highly suspected COVID-19 was analyzed (confirmed cases: total: 59, of which 37 IA). A special focus was put on the comparison of patients with an out-patient or in-patient management (16%) (59). Seventy-two percent of patients were receiving bDMARDs (anti-TNF, anti-IL17, anti-IL-23, and anti-IL-12/23 agents) or JAKi. Among hospitalized patients, comorbidities (hypertension, diabetes, or chronic obstructive pulmonary disease) were more common than in out-patients. Besides, oral GC (29 vs. 6%), HCQ (21 vs. 7%), and methotrexate (43 vs. 15%) were more frequently used in the former, whereas certain biologicals (mostly TNF-blockers and anti-IL12/23 agents) were more common in the latter group. Whether this indicates any possible protective effect of these biologics to prevent the occurrence of a CRS in RMD patients remains a matter of speculation.

Another possibility to learn about the course of COVID-19 on patients with RMD is to follow prospectively a cohort of patients with a defined disease and to note symptoms and outcomes of any SARS-CoV-2 infection. In a study from northern Italy with this objective, 320 patients with RA or SpA with an average age of 55 ± 14 years were interviewed and observed prospectively for 2 weeks (48). Among them, 13 patients had a confirmed or suspected SARS-CoV-2 infection or had been in contact with an infected person but had stayed asymptomatic. The use of bDMARDs and JAKi was heterogeneous (4 etanercept, 1 adalimumab, 1 tocilizumab, 2 abatacept, 2 tofacitinib, and 3 barictitinib). Almost half of patients were on regular hydroxychloroquine and/or low-dose glucocorticoid therapy. Only one patient, 65 years old, required hospital admission and was given oxygen and adjuvant therapy with antivirals and HCQ. All other patients paused their DMARDs for the time of the infection and had an uncomplicated course.

### Patients With Connective Tissue Diseases (CTD)

There have been only incidental reports of patients with CTD besides SLE: Of the few cases with systemic sclerosis (Table 2), the most striking common denominator is treatment with Rituximab, which seems to induce a late worsening of symptoms, leading to respiratory complications, also in patients without pre-existing lung disease (64, 84). Likewise, one patient from France with granulomatosis with polyangiitis, who was overweight and had hypertension under rituximab and corticosteroid therapy had a severe COVID-19 course with ARDS requiring intubation. The patient was discharged after 29 days.

The disease course of 17 French patients with SLE [median age: 53.5 (26.6–69.2)] was reported recently (62). Sixteen of the patients had quiescent rheumatic disease, the main comorbidities were obesity (59%) and chronic kidney disease (47%); 24% of them had secondary antiphospholipid syndrome (APS). All were on (different doses) of HCQ, 71% were taking glucocorticoids, most of them below 10 mg prednisone equivalent, and 41% took immunosuppressants. The clinical course of these patients was more severe than expected, 65% of them had signs of respiratory failure needing oxygen therapy. Almost half of these patients were admitted to intensive care and one third developed ARDS. One patient was treated with extracorporeal membrane oxygenation and two of the 17 patients died. Three patients developed acute renal injury; cardiac injury and venous thrombosis occurred in one patient each. An equally severe picture was displayed in five SLE patients from Michigan, were one patient died, and three needed mechanical ventilation (67). The reasons why these SLE patients had a worse outcome than those from other reports of the general population is unclear, however, long-term glucocorticoid treatment and comorbidities might have played a role. Additionally, an epigenetic dysregulation, i.e., DNA hypomethylation of ACE2 resulting in ACE2 overexpression in T cells of SLE patients, has been shown; this phenomenon could be perpetuated by the oxidative stress induced by viral infections or by SLE flares and thus could contribute to the worse disease course seen in this SLE cohort (94).

In contrast, Bozzalla et al. observed an overall mild course of COVID-19 in 12 patients of their cohort of 165 SLE patients, although the incidence of confirmed or suspected COVID-19 was higher in this cohort than in the general population of the area; nevertheless, this might be explained by a bias of higher testing (63).

A special aspect was observed in a 47-year-old Chinese SLE patient, who had been diagnosed with SLE 16 years earlier and had been on 7.5 mg of prednisone ever since, without other immunosuppression. She had experienced mild symptoms for 4 weeks and was diagnosed with COVID-19 7 weeks (87) after her presumed exposure to the virus having infected two family members in the meantime. At the time of diagnosis her nasopharyngeal swabs were negative, and she showed positive SARS-CoV-2 serology, suggesting that the symptoms experienced were part of the second phase. The unusually long incubation period and the supposed prolonged viral shedding might be due to the immunosuppression with GC which also warrants caution in other rheumatic patients on long term GC treatment.

Apart from case series, patient surveys have been carried out in CTD patients. The largest one comprised 916 patients in northeast Italy, of which 88% had underlying CTD (SLE, vasculitis, systemic sclerosis, myositis); 2.5% had a high suspicion of COVID-19, only two patients (0.21%) had a PCR confirmed infection, mortality was 0. The low prevalence of confirmed cases was similar to that of the general population of the Veneto. Overall, discontinuation of DMARD therapy was rare in these patients, with higher percentages in RA (10.8%) and SLE (3.3%) (91). A comparably low prevalence of COVID-19 was seen in other surveys from Milan and Belgium, possibly influenced by a high percentage of patients undertaking precautionary measures early in the pandemic (64, 65, 92, 93). Also in these surveys the ineffectiveness of HCQ as protection against COVID-19 was noted, as well as a negative effect of higher GC doses in terms of hospitalization and clinical features (anosmia/ageusia and diarrhea) (65).

In conclusion, data published in the first 6 months do not consistently describe a higher risk for infection with SARS-CoV-2 or a more severe course of COVID-19 in patients with either inflammatory arthritis or connective tissue diseases. However, due to early case series (62, 67) describing a more severe course of the infection in SLE patients, more data on COVID-19 from cohorts of SLE patients with confirmed SARS-CoV-2 infection are warranted.

### Antiphospholipid Syndrome (APS) and COVID-19

The thrombogenic nature of COVID-19 (17) is shared by rheumatic diseases, which should prompt further attention on RMD-associated coagulation problems such as the APS. Analogous to incidental reports of antiphospholipid antibodies (APL) in earlier coronavirus pandemics, these antibodies have occasionally been reported in COVID-19 (95), albeit of an IgA class, which have not yet been included in the classification criteria of APS (96). However, a role for IgA antibodies in clinical APS not meeting the serological criteria and SLE patients has been postulated (97, 98). Lupus anticoagulant (LAC) was present in 45% of a French cohort of 56 patients with COVID-19 without underlying RMD. In this group, 10% also tested positive for anti-cardiolipin (aCL) or anti-beta2glycoprotein1 (ab2gp1) antibodies (mostly associated with LAC) (99). Additional data from London corroborate the higher prevalence of LAC in COVID-19 patients. In 216 samples from COVID-19 patients screened for coagulation abnormalities in 24 h, 44 were found to have a prolonged aPTT, and 91% of the further investigated showed a positive LAC, often associated with reduced factor XII levels (100). Additionally, in a population of COVID-19 patients on the intensive care unit (ICU) 87% of the patients tested had a positive LAC (101). In contrast, antiphospholipid antibodies were commonly detected to a substantially lower degree with a preponderance of antibodies, which are not part of the official classification criteria for APS [IgA aCL; IgA ab2gp1; antibodies against phosphatidylserine/prothrombin (aPS/PT)] (102–104). Due to their disputed role in APS in general, the significance of these antibodies for COVID-19 is not yet clear. The occurrence of APL in viral infection is a well-known phenomenon, however, in most cases these are of IgM or IgG aCL subtypes with an antigen recognition differing from classical APS and are less thrombogenic in nature (105); in COVID-19 the latter finding has also been observed, casting a doubt on the clinical relevance of the characterized antibodies (102). Nevertheless, the pathogenicity of APL from COVID-19 patients was proven elegantly in a pre-publication by Zuo et al., in which IgG aPS/PT antibodies led to NETosis *in vitro*, thus contributing to thrombus formation, and increased thrombus extension *in vivo* in a mouse model (104). This finding is corroborated by the observation that APL occur preferably in critically ill patients (103). Altogether, the risk of thrombosis mediated by APS should be kept in mind and a prolonged aPTT should not prevent proper anticoagulation in patients. Besides, it needs to be tested whether the presence of the diverse APL is associated with an increased risk of worse outcomes in patients with COVID-19 (17) or *vice versa*, and if the emergence of LAC and APL in COVID-19 patients constitute a transient phenomenon or a persistently increased thrombogenic state.

Altogether, reports on patients with RMD infected with SARS-CoV-2 are still insufficient and are partly contradictory in terms of hospitalization and outcome. However, an unequivocal signal of higher risk for infection with SARS-CoV-2 or a more severe disease course of COVID-19 has not been detected. More data, especially larger cohorts of different patient groups are needed to provide a better risk stratification of RMD patients. Additionally, the question, whether patients receiving bDMARDs such as TNF-blockers or anti-IL-12/23 may be protected from a complicated disease course has to be clarified by future research. In conclusion, due to the generally increased susceptibility to infection, patients with rheumatic diseases should be considered at risk as long as the data remain scant.

## Does SARS-CoV-2 Increase the Risk of Developing a Rheumatic Disease?

Another yet unresolved question is whether COVID-19 might trigger autoimmune diseases. Although the detection of ANA has been reported in a third of COVID-19 patients in the acute stage (106), the persistence and clinical relevance thereof remains to be investigated.

Whereas, arthralgias and myalgias might be one of the symptoms of COVID-19, data on the incidence of RA or other IA after COVID-19 have not been published yet.

In a Korean study of 24,117 newly diagnosed RA patients, published before the discovery of SARS-CoV-2, an increased risk of developing rheumatoid arthritis after infection with other coronaviruses or influenza was reported. For an increase of 1% of coronavirus-infected patients, a 9.2% increase in the incidence of RA in the following months was observed, especially in women and older patients (107). Whether, there is direct molecular mimicry between coronavirus and host antigens or whether coronaviruses activate other, yet unclear mechanisms leading to chronic autoimmunity has to be investigated further.

Analogous to previous (small) peaks during SARS/MERS, a pediatric inflammatory multisystem syndrome displaying similarities with Kawasaki syndrome has been observed in association with SARS-CoV-2. The first child reported, a 6-month-old infant, received a single dose of 2 g/kg intravenous immunoglobulin (IVIG) and high dose acetylsalicylic acid and was discharged shortly thereafter (108). The unusual accumulation of eight children suffering from a disease reminiscent of Kawasaki syndrome with a combination of rash, conjunctivitis, peripheral oedema, extremity pain, and serious gastrointestinal symptoms prompted a national alert in Great Britain at the end of April (109). Although only two (one of them postmortem) of the children tested positive for SARS-CoV-2, all of them were reported to have antibodies. This suggests a SARS-CoV-2 triggered severe hyperinflammation necessitating the admission to the pediatric ICU, where one 14-year-old child eventually succumbed to a large cerebrovascular infarct. All children experienced either cardiac complications or serositis, were treated with inotropics, and apart from the mentioned 14-year-old are alive at this point. Since the first description, several larger case series have been published describing a clinical spectrum including fever, gastrointestinal symptoms, and rash as well as the complications of myocardial injury, shock, and development of coronary artery aneurysms (110–113). The disease associated with SARS-CoV-2 and the “classical” Kawasaki syndrome differ in terms of age, inflammation markers including ferritin, which are all higher in SARS-CoV-2, as well as more severe cardiac involvement (112, 113). An unusual and alarming treatment resistance to IVIGs was seen in more than 60% of a French cohort of 16 patients. This occurred especially in patients >5 years old with high ferritin levels and should prompt rapid escalation of immunosuppression with tocilizumab or anakinra to avoid serious cardiac sequelae (114). The exact relationship between the virus and the hyperinflammation has yet to be elucidated in order to optimize a treatment regimen.

## Should We Use Dmards for the Therapy of COVID-19?

Elevated levels of pro-inflammatory cytokines, including IL-6 are associated with severe COVID-19 and an increase in IL-6 is associated with increased mortality (11, 115). An excessive increase of C-reactive protein (CRP), IL-6, and ferritin observed in COVID-19 cases was termed CRS in analogy to similar findings in hemophagocytic lymphohistiocytosis and CAR-T cell therapy. Blockade of the IL-6 receptor (IL-6R) using tocilizumab is approved by the FDA to alleviate the CRS after hematological CAR-T cell therapy (116). In COVID-19 tocilizumab may be considered in patients with high levels of inflammatory markers such as IL-6 and worsening respiratory insufficiency, requiring non-invasive ventilation or intubation (117). However, evidence for these recommendations from randomized control trials (RCT) is lacking. Several reports have been published: Toniati et al. reported an improvement in 77 out of 100 patients treated with tocilizumab while 20 died (118). Xu et al. described 20 patients with severe COVID-19 and improvement of symptoms after a single administration of tocilizumab (119). Luo et al. reported 15 COVID-19 patients treated with tocilizumab, of whom three died (120). In addition, several retrospective case-control studies on the use of tocilizumab in COVID-19 have been published (121–126), of which a majority reported a favorable outcome of tocilizumab treatment compared to controls. Of these, Campochiaro et al. and Canziani et al. did not find a statistically reduced mortality in tocilizumab treated patients, although both studies showed a respective trend (121, 122) additionally, tocilizumab treated patients required invasive ventilation statistically less frequently than controls (122). In line with this observation the outcomes of several studies suggest that patients may benefit most from tocilizumab if given before invasive ventilation (125, 127). In a prospective open single-arm study investigating 63 COVID-19 patients with signs of CRS, administering tocilizumab within the first 6 days of hospitalization was associated with a better outcome than later in the course of disease, irrespective of the intravenous or subcutaneous route of administration (128). Preliminary results released of a large RCT investigating the use of the IL-6 receptor blocker sarilumab in COVID-19 are ambivalent^1^. Patients with critical COVID-19 treated with 400 mg of sarilumab tended to less likely need mechanical ventilation and showed clinical improvement more often than the placebo group. However, in patients with non-critical, severe COVID-19 the group receiving sarilumab trended toward unfavorable outcomes when compared to placebo. In this study, the patients had not been pre-stratified for high inflammatory burden. Thus, in the absence of a cytokine storm where IL-6 receptor blockade cannot be effective, adverse events may deteriorate outcomes. Bacterial superinfection, septic shock, gastrointestinal perforation, and viral myocarditis have been reported in COVID-19 after treatment with tocilizumab (118, 126, 129). In summary, IL-6 blockade may be a useful option in COVID-19 when administered early to those with signs of excessive inflammation. A clearer picture will emerge as randomized controlled studies for tocilizumab and sarilumab currently conducted in COVID-19 will be published.

The beneficial role of IL-1 blockade in life-threatening hemophagocytic lymphohistiocytosis (HLH) syndrome has been well-established (130). Due to the pathophysiological similarities pertaining to the macrophage activation between HLH and the hyperinflammation seen in the second phase of COVID-19, a small number of patients have been treated with the IL-1 receptor antagonist anakinra. Two case series report anakinra treatment in 9 and 11 patients, respectively (131, 132). Eight out of nine and seven out of 11 patients treated with anakinra did not require invasive ventilation. Besides a slight elevation of transaminases, anakinra was well-tolerated. However, it is unclear if the patients might have improved without the blockade of IL-1. Cavalli et al. conducted a retrospective case-control study of 29 COVID-19 patients treated with a high dose of intravenous anakinra (10 mg/kg/day) comparing their outcome to seven patients receiving 100 mg of anakinra subcutaneously twice daily and 16 controls on standard treatment (133). All patients suffered from moderate-to-severe ARDS and hyperinflammation. While subcutaneous application of anakinra was terminated due to lack of efficacy, survival in those receiving high dose anakinra treatment was superior to those receiving standard care (90 and 56%, respectively). Although pathophysiologically reasonable, solid evidence from randomized control trials are needed; in this respect at least 10 trials are underway targeting IL-1 in COVID-19.

Corticosteroids have a broad anti-inflammatory potential and are readily available. Therefore, they have been considered for use in critical COVID-19. High-dose corticosteroids at admission were identified as a risk factor for COVID-19 mortality, and they are not recommended by the WHO for viral pneumonia (11)^2^. In a small observational study from China the use of corticosteroids was not associated with shorter hospitalization or more rapid virus clearance (134). Still, corticosteroids in variable doses are currently used in critical COVID-19 patients, especially those with ARDS (117). This practice is supported by data from the British RECOVERY trial, a randomized, controlled, open-label study including more than 6,000 hospitalized COVID-19 patients (135). A daily dose of 6 mg dexamethasone reduced 28-day mortality by one third in patients receiving invasive mechanical ventilation at randomization. Patients requiring oxygen without invasive mechanical ventilation did benefit from the addition of dexamethasone to a lesser extent, while those not receiving respiratory support of any kind did not benefit at all. In addition, ARDS patients were reported to develop glucocorticoid resistance requiring excess corticosteroids to overcome progressive inflammation (136). This, suggests that dexamethasone is important in the later stage of infection, characterized by CRS, and ARDS, whereas in the first stage, immunosuppression might not be of additional value (137).

The antimalarial drugs chloroquine (CQ) and hydroxychloroquine (HCQ) have been suggested as potential antiviral agents in COVID-19. The rationale arises from the observation that CQ and HCQ can prevent SARS-CoV-2 replication *in vitro* probably by increasing endosomal pH or increasing intracellular zinc levels (61, 138).

*In vivo*, chloroquine did not reduce SARS-CoV viral titers in a mouse model (139). Early clinical trials from China, suggested improved clinical outcomes of COVID-19 when treated with chloroquine as compared to controls groups (1, 140). In a controversial French study, 26 patients were treated with HCQ and azithromycin and compared to a cohort receiving standard therapy. Viral load decreased more rapidly in the HCQ and azithromycin group. However, due to methodological shortcomings the results should be interpreted with caution (141). Two large observational studies could not confirm these results (142, 143). In a retrospective analysis of 1,376 propensity-score-matched COVID-19 patients, the rate of intubation or death was similar in those receiving HCQ or standard care (143). Moreover, a retrospective study comparing mortality of HCQ/CQ to standard therapy found higher mortality in those receiving HCQ/CQ (144). In order to find the optimal anti-viral dose of HCQ, a RCT compared two doses of CQ (600 mg bid for 10 days or 450 mg bid on day 1 and once daily for 4 days) in 81 patients with COVID-19 (145). The higher dose of HCQ resulted in an increased lethality. Furthermore, on electrocardiogram QTc-intervals >500 ms were found in 19 and 11 percent in the high- vs. low-dose group. Other studies reported QTc-intervals >500 ms in 5–23% of the patients treated with HCQ and 0–33% in those with azithromycin co-treatment (146–149). Other adverse events of HCQ and azithromycin co-treatment reported in 2.4% were mild (146). In June 2020 an interim analysis of the hydroxychloroquine arm of the RECOVERY trial revealed no benefit of HCQ compared to standard of care (150). A total of 1,542 patients had been randomized to hydroxychloroquine and compared with 3,132 patients receiving standard of care alone. No significant difference in the primary endpoint of 28-day mortality could be found. In summary, evidence for clinical efficacy of CQ and HCQ in COVID-19 is limited. Therefore, antimalarial drugs should not be used in COVID-19 outside clinical trials; accordingly, the FDA revoked the emergency use authorization of these antimalarials in June 2020 and issued a cautionary statement in July (151).

Baricitinib was identified as a potential therapeutic agent for COVID-19 employing a drug repurposing software. The suggested mechanism is specific for baricitinib, not constituting a class-effect of JAKi, and works through the inhibition of virus uptake by the blockade of endocytosis (152). In line with these considerations, a retrospective study compared 173 COVID-19 patients receiving baricitinib to 78 patients on standard therapy (153). Case fatality and intensive care admission was significantly less frequent in the patients receiving baricitinib. Additionally, a higher proportion of patients had negative nasopharyngeal swabs on discharge in the baricitinib arm. Altogether, randomized controlled trials for the use of baricitinib in COVID-19 seem worthwhile and are ongoing.

Colchicine, used in gout due to its effects on the chemotaxis of neutrophils and monocytes, has been proposed as a potential anti-inflammatory therapy in COVID-19 since recruitment of neutrophils is a key factor in severe COVID-19 pneumonia (154). Evidence from a Greek randomized, controlled trial on 105 COVID-19 patients suggests a benefit of colchicine treatment (155). However, the primary outcome measures were focused on inflammatory markers (CRP and high sensitivity cardiac troponin) and overall clinical deterioration, which may have been biased by the open-label design. Larger trials with mortality or invasive ventilation as endpoints are needed to support the findings of this initial trial.

Intravenous gammaglobulins (IVIG) serve as an anti-inflammatory rescue therapy in autoimmune diseases although the precise mode of action is elusive. In addition, commercially available IVIG preparations contain antibodies cross-reactive to SARS CoV-2 or endogenous proteins, such as cytokines (156). Therefore, IVIG have been used in COVID-19 (157–159). However, as no case-control studies or RCT's have been published so far, the therapeutic efficacy of IVIG in COVID-19 is unknown.

## Conclusion

Overall, data regarding COVID-19 and RMD are sparse. An increased risk of developing COVID-19 or a complicated course of COVID-19 cannot be deduced from data on COVID-19 or other infections in patients with inflammatory arthritis. Further studies are needed to investigate the course of COVID-19 in connective tissue diseases such as SLE or vasculitis, particularly in patients with already existing organ damage and/or other comorbidities. A further point of consideration is the role of bDMARDs or JAKi. However, more data are needed to disentangle a possible protective role of biological DMARDs or JAKi against a possible CRS as compared to an increased susceptibility for viral infections (summarized in Box 2).

Box 2 **Overview of open and resolved research questions.**

**Issues needing further research and data:**

- is there a role for Antiphospholipid antibodies in the thrombogenicity of COVID-19
- is there a protective role of JAKi, IL-6, bDMARDs
- if yes, when should they be applied
- is the risk of a complicated course of CTD/ vasculitis patients heightened?

**Issues that have been answered**

- there is no protective role of HQ in RMD patients
- RMD patients can continue most of their DMARDs without problems
- there is no increased risk for a complicated course of COVID-19 in patients with inflammatory arthritis.

## Author Contributions

All authors critically evaluated the literature and wrote the paper.

## Conflict of Interest

The authors declare that the research was conducted in the absence of any commercial or financial relationships that could be construed as a potential conflict of interest.

## Footnotes

^1^Available online at: https://www.prnewswire.com/news-releases/regeneron-and-sanofi-provide-update-on-us-phase-23-adaptive-designed-trial-of-kevzara-sarilumab-in-hospitalized-covid-19-patients-301047326.html [press release].

^2^Available online at: https://apps.who.int/iris/bitstream/handle/10665/331446/WHO-2019-nCoV-clinical-2020.4-eng.pdf
